# Prospective Comparison of Moderate Conscious Sedation and Anesthesia Assistance for the Performance of Endoscopic Retrograde Cholangiopancreatography (ERCP)

**DOI:** 10.1155/2021/8892085

**Published:** 2021-04-13

**Authors:** Ahmed Akhter, Ravi Patel, Eric Nelsen, Mark E. Benson, Deepak V. Gopal, Anurag Soni, Patrick Pfau

**Affiliations:** Section of Gastroenterology and Hepatology, Department of Medicine, University of Wisconsin, School of Medicine and Public Health, Madison, WI, USA

## Abstract

**Objectives:**

Recent trends have favored the use of anesthesia personnel more frequently for advanced endoscopic procedures. We hypothesize a selective sedation approach based on patient and procedural factors using either moderate conscious sedation (MCS) or general anesthesia (GA) will result in similar outcomes and safety with significant cost savings.

**Methods:**

A 12-month prospective study of all adult endoscopic retrograde cholangiopancreatography (ERCPs) performed at a tertiary medical center was enrolled. Technical success, cannulation rates, procedural related complications, procedure time, and cost were compared between MCS and GA.

**Results:**

A total of 876 ERCPs were included in the study with 74% performed with MCS versus 26% with GA. The intended intervention was completed successfully in 95% of cases with MCS versus 96% cases with GA (*p* = 0.59). Cannulation success rates with MCS were 97.5 versus 97.8% with GA (*p* = 0.81). Overall, adverse event rates were similar in both groups (MCS: 6.6% vs. GA: 9.2%, *p* = 0.21). Mean procedure time was less for MCS versus GA, 18.3 and 26 minutes, respectively (*p* < 0.0001). Selective use of MCS vs. universal sedation with GA resulted in estimated savings of $8,190 per case and $4,735,202 per annum.

**Conclusions:**

Preselection of ERCP sedation of moderate conscious sedation versus general anesthesia based upon patient risk factors and planned therapeutic intervention allows for the majority of ERCPs to be completed with MCS with similar rates of technical success and improvement in resource utilization and cost savings compared to performing ERCPs universally with anesthesia assistance.

## 1. Introduction

Endoscopic retrograde cholangiopancreatography (ERCP) requires adequate sedation for successful completion. The degree of complexity of ERCPs can vary considerably and increased complexity may lead to longer procedure times, decreased technical success, and increased adverse events. The American Society for Gastrointestinal Endoscopy recommends anesthesia provider-administered sedation be considered for complex endoscopic procedures [1, 2]. There is a paucity of data regarding the type of sedation that should be used for ERCPs and specifically the role of moderate conscious sedation in advanced endoscopic procedures. The universal use of anesthesia for ERCPs has greatly increased with little data to support this practice as compared to traditional moderate conscious sedation (MCS). The majority of studies examining ERCP sedation have concentrated on comparing general anesthesia (GA) to monitored anesthesia care (MAC) while ignoring the role of MCS. Despite the marked trend towards anesthesia administered sedation for ERCP, current standards of care have not adopted a generalized practice of GA or MAC for all ERCPs [3]. The aim of this study is to determine if a selective sedation approach based on patient and procedural factors using either MCS or GA will result in similar rates of technical success and adverse events while offering more favorable utilization of resources with cost savings compared to universally performing all ERCPs with anesthesia administered sedation.

## 2. Materials and Methods

This was a prospective study of all adult patients undergoing ERCP performed at a single tertiary care center over a 12-month period. Patients consented prior to the procedure and data was collected and entered after completion of each procedure. Patients were excluded if an additional procedure was performed along with the ERCP and/or if it required the assistance of an interventional radiologist or surgeon. Patient data recorded at the time of the ERCP included age, gender, BMI, indication for ERCP, and American Society of Anesthesiologists (ASA) class [4]. Procedural characteristics included the Modified Schutz Score, the type of intervention performed, presence of a native papilla, and total procedure, operating room (OR), and recovery time were recorded [5]. All ERCPs were performed among four trained advanced endoscopists with at least 5 years of experience with an advanced endoscopy fellow. The study was approved by the institutional review board of the University of Wisconsin.

The type of sedation was directed by the endoscopist performing the procedure (GA vs. MCS) based upon factors including BMI, ASA class, complexity of ERCP, cardiopulmonary comorbidity, medication history/substance abuse, and prior sedation administered [5]. Anesthesia-directed sedation for ERCP was performed with GA and no cases were performed via MAC due to institutional preference. Patients were sedated under the supervision and direction of an attending anesthesiologist. MCS was administered by endoscopy nurses trained in providing sedation under the endoscopist supervision with a combination of fentanyl, midazolam, and diphenhydramine. All patients were positioned in the prone position except if body habitus or musculoskeletal abnormalities precluded such positioning.

Primary outcomes evaluated were cannulation rates, successful completion of the intended procedure, total procedure time, OR time (time spent in endoscopy room), and recovery time for patients who underwent GA versus MCS. In addition, adverse events including post-ERCP pancreatitis, SRAE, perforation, bleeding, unplanned hospital admission, and mortality related to the procedure were compared between patients who underwent GA and those who underwent MCS. Failure of MCS and conversion or subsequent need for GA to complete ERCP were recorded. Post-ERCP pancreatitis was defined using the revised Atlanta classification [6]. SRAE were defined as any event that caused procedural interruption or premature termination of the procedure including sedation intolerance, hypoxia, hypotension, arrythmia, and respiratory failure. Major bleeding was defined as patients who required blood transfusion or hospital admission for melena within 30 days of ERCP. Every outpatient case was contacted the day after the procedure by phone with cases performed on Fridays being contacted on Mondays with recording of any complications or adverse events related to the procedure. Longer-term follow-up was conducted with a review of the electronic medical chart for 30 days after ERCP. Review of emergency room visits or relevant telephone or nursing-related encounters was reviewed for evaluation related to postprocedural complications.

We compared the utilization of resources between patients who underwent GA and those who underwent MCS as measured by professional and facility charges. University of Wisconsin financial services calculated the mean anesthesia provider charge for ERCPs provided with GA. We also calculated the mean anesthesia facility fee for use of general anesthesia for ERCPs. The facility fee includes the cost of the room, nurse, medications, supplies, and recovery. We then calculated the mean provider and facility fee for patients in whom ERCP was performed with MCS. We calculated the difference between charges for anesthesia-assisted ERCP and those performed with MCS on a per-case and per-year basis. For the per-year calculation, we also considered the MCS patients who failed MCS and had to have a repeat ERCP done with GA.

Procedure, OR, and recovery times were reported as mean times and categorical comparisons were made using the Chi-square test. A *p* value of <0.05 was considered statistically significant. Utilization charges were calculated using the average anesthesia professional and facility charge, sedation medications and supplies, fluoroscopy expense, recovery charge, and procedure cost of an ERCP with bile duct cannulation and stent exchange.

## 3. Results

A total of 876 patients were included in the study with 74% undergoing ERCP with MCS and 26% with GA. Patients who were selected for GA had a female predominance, were younger, were more obese, and had a greater percentage of previous biliary endoscopic intervention as compared to patients who were chosen for MCS (Table 1). The Modified Schutz Score measuring procedure difficulty was higher for patients who underwent ERCP with GA as compared to MCS along with a higher ASA class (Table 2). The median complexity of all ERCPs performed was 2/4 based upon the complexity scale as defined by the Modified Schutz Score [5]. Indications for procedures with MCS and GA are listed in Table 2. For moderate conscious sedation, the mean fentanyl dose was 179.8 mcg (range 50–400); mean midazolam dose 7.2 mg (range 2–21); and diphenhydramine 25.9 mg (0–50).

**Figure 1:**
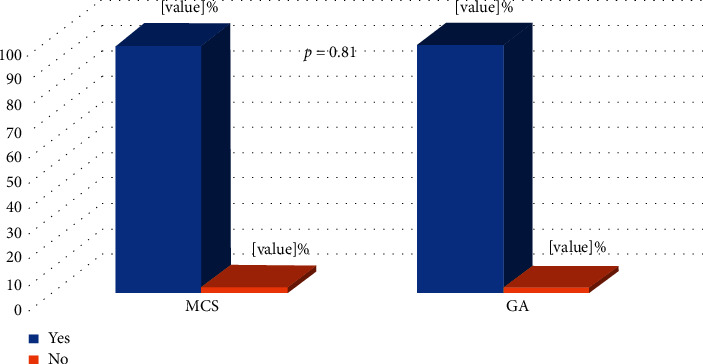
Percent of cannulation success between those patients who underwent MCS and those who underwent GA.

**Figure 2:**
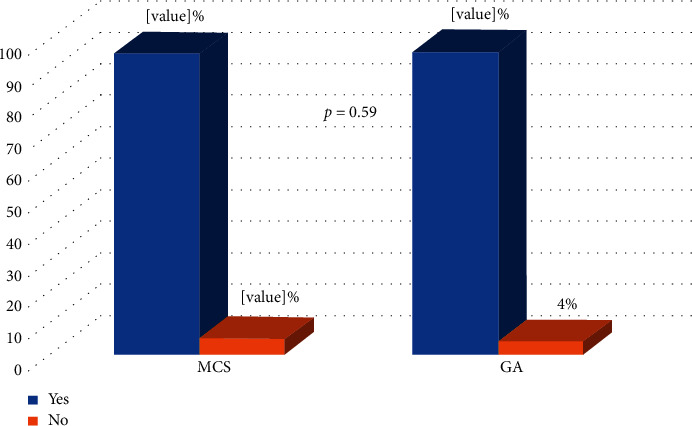
Percent of overall technical and clinical success of cases between those patients who underwent MCS and those who underwent GA.

There was no significant difference in cannulation success between groups (MCS: 97.5% vs. GA: 97.8%) (Figure 1). Primary outcomes including completion rates of the intended intervention were not significantly different between those patients selected for MCS versus GA (95.2% vs. 96%) (Figure 2). Adverse events were not significantly different between both groups (MCS: 6.6% vs. GA: 9.2%) (Table 3). No reversal agents were administered to the patients in the MCS group during the course of the study. There were no deaths related to ERCP adverse events up to 30 days after the procedure.

There were 31 unsuccessful cases in patients who underwent MCS with 52% (16/31) of those due to inadequate sedation or SRAE for which the case was stopped before completion of the desired intervention. Failure of sedation was related to adverse events (SRAE) which was defined as any event that caused procedural interruption or premature termination of the procedure including hypoxia, hypotension, arrhythmia, and respiratory failure in 15 of 16 failed cases. Only one failed case was solely secondary to sedation intolerance. The remaining patients (15/31) in which a successful outcome was not able to be achieved with MCS were attributed to anatomical or technical factors such as altered anatomy, failed cannulation, or inability to complete desired therapeutic intervention. Overall, 4.8% of all patients (31/647) who underwent ERCP with MCS were unsuccessful due to inability to tolerate MCS and adequately sedate the patient or because of purely technical failure such as failed cannulation. 3.4% of patients (22/647) who failed MCS because of sedation or technical reasons were reattempted with GA. 4% (9/229) of cases with GA were unsuccessful and none were attributed to SRAE.

Mean OR time was shorter in patients who underwent MCS versus GA, 50.8 versus 54.5 minutes (*p* = 0.01). The mean procedure time for patients who underwent ERCP was less with MCS, 18.3 minutes versus 26 minutes for those sedated with GA (*p* < 0.0001). The mean time in recovery was shorter in those who underwent MCS versus GA, being 80.7 versus 91.8 minutes (*p* < 0.001). The majority of patients sedated with MCS had mean procedure times that were ≤15 minutes (56%) with 88% of cases sedated with MCS lasting 30 minutes or less (Table 4).

The mean facility charge of an ERCP performed with general anesthesia personnel was $20,688 and the mean facility fee for ERCP with MCS was $14,093 with a difference per case of $6,595. The additional professional anesthesia fee for ERCP cases was $1,595. The overall additional costs of the cases performed with GA included the additional facility fee ($6,595) and anesthesia professional fee ($1,595) totaled $8,190 per case.

Per annum, 647 cases were performed with MCS and 229 cases were performed with GA. If all ERCPs were performed with GA additional charges per year, it would have totaled $5,298,930 over the course of the year. In the year of the study 22 ERCPs that failed with MCS being repeated with GA an estimated charge of 22°×°($ 20,688 + 1,595 + 3,343 (mean endoscopist physician fee)) = $563,728. Thus, the overall cost of savings of using selective MCS rather than universal GA sedation decreased charges by $4,735,202.

## 4. Discussion

ERCPs are increasingly being performed for therapeutic indications with varying complexity and relatively low endoscopic related complications. The length of the procedure and the complexity of the intended therapeutic intervention make effective sedation an integral part of achieving technical success. There has been an increasing trend towards endoscopic procedures being performed with MAC or GA provided by a trained anesthesia professional [7–9]. Most endoscopic procedures, including the patients in this study, are performed with anesthesia services on those who are considered low risk (ASA class 1 or 2) [10]. The American Society of Gastrointestinal Endoscopy (ASGE) suggests anesthesia provider-administered sedation can be advantageous for ERCPs [1]. However, there is limited data to support the recommendation of universal GA for ERCPs nor the rapidly increasing trend wherein the vast majority of ERCPs are performed with anesthesia assistance.

The most common approaches for sedation of patients undergoing ERCP are the use of GA or MAC with an increasing number of studies comparing these two sedation modalities rather than a comparison of anesthesia assistance versus MCS. In a study comparing GA versus MAC for patients at high risk for sedation related adverse events (SRAE), the incidence of SRAE was found to be lower with the use of GA versus MAC (9.9% vs. 51.5%) with 10% of patients in the MAC group being converted to GA [11]. A study of 528 consecutive patients undergoing ERCP with GA or MAC found SRAE in 21% of patients, with MAC; there was a higher incidence of hypoxia and GA patients had more cardiovascular events [12]. Another prospective study of 393 patients also found MAC to be safe and feasible for patients undergoing ERCP [13]. Prior studies have shown similar rates of SRAE of 15–24% for patients undergoing anesthesia administered sedation with the caveat that they rarely require premature termination of the procedure [14, 15]. Analysis of 27,000 ERCPs found MAC versus GA had similar rates of adverse events; however, SRAEs were lower with anesthesia personnel when compared to endoscopist-administered sedation [16].

A study analyzing 3058 patients who underwent ERCP with MCS found 4% of patients required reversal agents. Reversal agent use was associated with 6% morbidity [17]. A retrospective study of 1,056 ERCPs found 14% of ERCPs were aborted in patients who underwent MCS mainly due to inadequate sedation compared to a failure rate of 7% with GA [18]. Vargo et al. demonstrated adverse events ranging from 8 to 18.9% for patients undergoing MCS or MAC with no significant difference between either group [15]. A review of four RCTs comparing MCS versus propofol-based sedation found no difference in mortality or cardiorespiratory complications [19, 20]. However, nonanesthesia personnel were involved in administering sedation in all the studies analyzed which is distinctly different from the present practice where anesthesia personnel provide sedation [20].

The paucity and heterogeneity of data regarding the effectiveness and safety profile of MCS versus MAC/GA for patients undergoing ERCPs establish a need for more precise guidelines regarding the extent to when anesthesia services should be involved. Our purpose was to determine if preselection of sedation (MCS vs. GA) based upon patient risk factors and intended therapeutic intervention would result in a similar safety and effectiveness profile along with favorable utilization of resources. Posed in a different way, we sought to answer the question of can MCS still be used safely and effectively in the majority of ERCPs or should all ERCPs be performed with anesthesia assistance?

We found no difference in cannulation or technical success in patients preselected for MCS versus GA. Cannulation success did not differ despite the significant predominance of a native papilla in the MCS compared to those who underwent GA. “Native papilla may theoretically increase the risk of technical failure or postprocedural complications such as post-ERCP related pancreatitis. However, the presence of a native papilla does not directly impact risks related to sedation. Our results demonstrate that despite the predominance of the native papilla in the MCS, our overall rates of endoscopic success were in line with rates of technical success achieved in patients who underwent either MAC or GA [10, 11].” Only 3.4% of patients who underwent MCS required a reattempt with GA. Overall, adverse events were not significantly different between patients who underwent MCS versus GA. The low MCS ERCP sedation failures and equivalent adverse events between MCS and GA are likely because we preselected the most ill, high risk, and challenging patients for GA leaving the healthier patients and less challenging cases for MCS.

Patients who underwent MCS had shorter procedure time, shorter time in the procedure room, and shorter recovery time when compared to those who underwent GA. This is not surprising because the two groups were not meant to be equal with more complex cases that have the potential to last longer were sedated with GA. A closer examination of procedure time makes a stronger argument for selective use of MCS for ERCPs rather than universal use of anesthesia. Over half of the MCS cases lasted 15 minutes or less and 88% lasted 30 minutes or less. It is difficult to justify using general endotracheal intubation with an anesthesiologist and the associated resources and cost for cases that will almost always last 30 minutes or less and even less so when half of these cases last 15 minutes or less.

Total cost savings based upon preselection of sedation resulted in savings of almost five million dollars in a fiscal year based upon additional charges that would have been incurred if GA would have been performed for all patients. It is important to note this was slightly mitigated with the cost required to reattempt ERCPs with GA and extended hospital stays in the minority of patients for whom technical success was not achieved with MCS. Besides the strict savings in cost, selective sedation of the majority of patients with MCS is beneficial if anesthesia resources are limited at an institution allowing anesthesia personnel to work in other needed areas of the healthcare facility.

Overall, our rates of endoscopic success were in line with rates of technical success achieved in patients who underwent MAC or GA [10, 11]. SRAE in prior trials regardless of the type of sedation is estimated to range between 7 and 20% [10, 11, 21]. SRAE in our study accounted for 2.5% of all cases. This may in part be explained by prior studies defining SRAE to include those patients for whom airway maneuvers would be required (chin lift, jaw thrust, etc.) without termination of the procedure. We did not include airway maneuvers such as chin lift as a significant SRAE as these types of maneuvers can be considered a standard part of maintaining appropriate oxygenation for all patients undergoing sedation [22]. In addition, the use of low volume intravenous fluids or occasional injections of vasopressors during cases with GA, particularly after induction, was not considered a SRAE if they did not require procedural interruption and did not affect patient outcome. SRAE may also be lower given our procedure times are lower than the previously published studies with a mean procedure time <20 min for patients undergoing MCS as procedure time is associated with a greater risk of adverse events [12]. Finally, and likely most importantly, our SRAE are lower because of how our study was designed. The most ill, obese, and complex cases were placed in the GA group leaving the MCS group with healthier patients and less complex ERCPs.

Our study was not intended to be a direct comparison of MCS to GA sedation. Our aim was to determine if the majority of patients preselected as low risk could have successful outcomes with improved cost efficiency when using MCS. A potential weakness of our study is that there was not a hard-defined preprocedure set of criteria for GA versus MCS and the risk of procedure and complexity with the need for GA were determined by the endoscopist. However, our results showing higher BMI, higher ASA class, and higher complexity as defined by the Modified Schutz Score demonstrate that we did successfully select the higher risk patients for sedation with GA. Further, we acknowledge this study is at risk for a beta error given the low rate of complications after ERCP. The study also has limitations due to the heterogeneity of the patients within each study cohort. We have tried to minimize this risk by increasing our study size and comparing patients with similar demographics drawn from the same population.

## 5. Conclusions

Our study demonstrates preselection of ERCP sedation based upon patient risk factors and planned therapeutic intervention may allow for a significant proportion of patients to be sedated without the use of anesthesia assistance. MCS can be used to sedate the majority of patients undergoing ERCP with significant savings in cost and resources. However, given the increased use of anesthesia assistance for all procedures without substantive evidence, more data and comparative trials are needed of MCS versus GA for advanced endoscopic procedures to determine the precise algorithm used to guide the appropriate sedation approach.

## Figures and Tables

**Table 1:** Patient demographics of those who underwent moderate conscious sedation and general anesthesia.

Patient characteristics	MCS *n* = 647	GA *n* = 229	*p* value
Mean age (range)	62.2 (20–95)	53.7 (9–92)	<0.0001
Mean BMI (range)	28.4 (14.2–55.3)	30.0 (14–63)	0.004
Male (%)	391 (60.4%)	115 (50.2%)	0.007
Median ASA class (range)	2 (1–4)	3 (2–5)	*—*
Native papilla (%)	329 (50.9%)	84 (36.7%)	0.0002

MCS: moderate conscious sedation; GA: general anesthesia; BMI: body mass index; ASA: American Society of Anesthesiologists.

**Table 2:** Procedural complexity and indications of patients who underwent moderate conscious sedation and general anesthesia.

Patient characteristics	MCS *n* = 647	GA *n* = 229	*p* value
*Median Modified Schutz Score (1–4)*	*2 (1–4)*	*2 (1–4)*	*—*
*Median ASA class (range)*	*2 (1–4)*	*3 (2–5)*	*—*
Indications:			
1. Choledocholithiasis	201 (31%)	59 (26%)	*0.152*
2. Cholangitis	51 (8%)	19 (8%)	*0.887*
3. Malignancy	160 (25%)	20 (9%)	*<0.001*
4. Postliver transplant	106 (16%)	33 (14%)	*0.529*
5. Bile leak/pancreas fistula	52 (8%)	39 (17%)	*<0.001*
6. Other (PSC, sphincter of Oddi dysfunction, ampullectomy, pancreatitis, etc.)	77 (12%)	59 (26%)	*<0.001*

MCS: moderate conscious sedation; GA: general anesthesia; ASA: American Society of Anesthesiologists; PSC: primary sclerosing cholangitis.

**Table 3:** Overall complications and sedation-related adverse events in patients who underwent moderate conscious sedation and general anesthesia.

Adverse events	MCS (*n* = 43)	GA (*n* = 21)
Post-ERCP pancreatitis	19 (2.9%)	7 (3.1%)
Major bleeding	5 (0.8%)	1 (0.4%)
Sedation related adverse events (i.e., respiratory failure, hypotension, and arrhythmia)	11 (1.7%)	1 (0.4%)
Tear/perforation	1 (0.2%)	2 (0.9%)
Other (infection, pain, stent migration, etc.)	7 (1.1%)	10 (4.4%)
Deaths	0	0

MCS: moderate conscious sedation; GA: general anesthesia; ERCP: endoscopic retrograde cholangiopancreatography.

**Table 4:** Procedure time between patients who underwent moderate conscious sedation and general anesthesia.

Actual procedure time	MCS *N* = 647	GA *N* = 229
≤15 minutes	361 (56%)	93 (41%)
16–30 minutes	207 (32%)	75 (33%)
31–59 minutes	70 (11%)	42 (18%)
≥60 minutes	9 (1%)	19 (8%)

MCS: moderate conscious sedation; GA: general anesthesia.

## Data Availability

All data are present in results and tables. Further inquiries about data can be obtained from the corresponding author.

## References

[1] Early D. S., Lightdale J. R., Vargo J. J. (2018). Guidelines for sedation and anesthesia in GI endoscopy. *Gastrointestinal Endoscopy*.

[2] Edgcombe H., Carter K., Yarrow S. (2008). Anaesthesia in the prone position. *British Journal of Anaesthesia*.

[3] Care ASOATFOP (2002). Practice guidelines for postanesthetic care: a report by the American society of anesthesiologists task force on postanesthetic care. *Anesthesiology*.

[4] Hurwitz E. E., Simon M., Vinta S. R. (2017). Adding examples to the ASA-physical status classification improves correct assignment to patients. *Anesthesiology*.

[5] Cotton P. B., Eisen G., Romagnuolo J. (2011). Grading the complexity of endoscopic procedures: results of an ASGE working party. *Gastrointestinal Endoscopy*.

[6] Thoeni R. F. (2012). The revised Atlanta classification of acute pancreatitis: its importance for the radiologist and its effect on treatment. *Radiology*.

[7] Rodriguez H. J., Ghassemi K., Vesga L., Verma R. V., Bagatelos K. C., Ostroff J. W. (2006). Changes in sedation trends for ERCP before and after the droperidol black box warning. *Gastrointestinal Endoscopy*.

[8] Inadomi J. M., Gunnarsson C. L., Rizzo J. A., Fang H. (2010). Projected increased growth rate of anesthesia professional-delivered sedation for colonoscopy and EGD in the United States: 2009 to 2015. *Gastrointestinal Endoscopy*.

[9] Liu H., Waxman D. A., Main R., Mattke S. (2012). Utilization of anesthesia services during outpatient endoscopies and colonoscopies and associated spending in 2003-2009. *JAMA*.

[10] Predmore Z., Nie X., Main R., Mattke S., Liu H. (2017). Anesthesia service use during outpatient gastroenterology procedures continued to increase from 2010 to 2013 and potentially discretionary spending remained high. *American Journal of Gastroenterology*.

[11] Smith Z. L., Mullady D. K., Lang G. D. (2018). A randomized-controlled trial evaluating general endotracheal anesthesia versus monitored anesthesia care and the incidence of sedation-related adverse events during ERCP in high-risk patients. *Gastrointestinal Endoscopy*.

[12] Berzin T. M., Sanaka S., Barnett S. R. (2011). A prospective assessment of sedation-related adverse events and patient and endoscopist satisfaction in ERCP with anesthesiologist-administered sedation. *Gastrointestinal Endoscopy*.

[13] Barnett S. R., Berzin T., Sanaka S., Pleskow D., Sawhney M., Chuttani R. (2013). Deep sedation without intubation for ERCP is appropriate in healthier, non-obese patients. *Digestive Diseases and Sciences*.

[14] Paspatis G. A., Manolaraki M. M., Vardas E., Theodoropoulou A., Chlouverakis G. (2008). Deep sedation for endoscopic retrograde cholangiopancreatography: intravenous propofol alone versus intravenous propofol with oral midazolam premedication. *Endoscopy*.

[15] Vargo J. J., Zuccaro G., Dumot J. A. (2002). Gastroenterologist-administered propofol versus meperidine and midazolam for advanced upper endoscopy: a prospective, randomized trial. *Gastroenterology*.

[16] Smith Z. L., Markollari V., Nickel K. B., Olsen M. A., Vargo J. J., Kushnir V. (2018). 937 mode of sedation and serious adverse events during ercp: analysis of the clinical outcomes research initiative national endoscopic database (CORI-NED). *Gastrointestinal Endoscopy*.

[17] Papachristou G. I., Gleeson F. C., Papachristou D. J., Petersen B. T., Baron T. H. (2007). Endoscopist administered sedation during ERCP: impact of chronic narcotic/benzodiazepine use and predictive risk of reversal agent utilization. *The American Journal of Gastroenterology*.

[18] Raymondos K., Panning B., Bachem I., Manns M. P., Piepenbrock S., Meier P. N. (2002). Evaluation of endoscopic retrograde cholangiopancreatography under conscious sedation and general anesthesia. *Endoscopy*.

[19] Garewal D., Powell S., Milan S. J., Nordmeyer J., Waikar P. (2012). Sedative techniques for endoscopic retrograde cholangiopancreatography. *The Cochrane Database of Dystematic Reviews*.

[20] Thosani N., Banerjee S. (2013). Deep sedation or general anesthesia for ERCP?. *Digestive Diseases and Sciences*.

[21] Buxbaum J., Roth N., Motamedi N. (2017). Anesthetist-directed sedation favors success of advanced endoscopic procedures. *American Journal of Gastroenterology*.

[22] Vargo J. J. (2010). Anesthesia-mediated sedation for advanced endoscopic procedures and cardiopulmonary complications: of mountains and molehills. *Clinical Gastroenterology and Hepatology*.

